# Hypertension-Induced Thrombotic Microangiopathy Leading to End-Stage Renal Disease

**DOI:** 10.7759/cureus.33890

**Published:** 2023-01-17

**Authors:** Angel Juarez, Lidice Galindo, Maryam Gondal

**Affiliations:** 1 Department of Internal Medicine, Grand Strand Medical Center, Myrtle Beach, USA; 2 Department of Nephrology, Grand Strand Medical Center, Myrtle Beach, USA

**Keywords:** microangiopathic autoimmune hemolytic anemia, tma, hypertensive emergency, thrombotic microangiopathy, end-stage renal disease

## Abstract

Thrombotic microangiopathy (TMA) is a term used for a group of rare and life-threatening hematological conditions. Usually, these disease processes are characterized by microangiopathic hemolytic anemia (MAHA), thrombocytopenia, and microthrombi leading to tissue or organ injury. We present a case of a 41-year-old male with TMA induced by uncontrolled hypertension leading to end-stage renal disease requiring hemodialysis. Our goal is to highlight the importance of distinguishing hypertension-induced thrombotic microangiopathy from other etiologies of TMA, particularly thrombotic thrombocytopenic purpura (TTP), and its effect on renal function despite treatment focused on blood pressure control. Thus, it is a challenging diagnosis for clinicians to determine whether to initiate plasmapheresis for prompt treatment of suspected TTP in the setting of severe hypertension with thrombocytopenia.

## Introduction

Thrombotic microangiopathy (TMA) is a term used for a group of disorders that are rare and life-threatening. Usually, these disease processes are characterized by microangiopathic hemolytic anemia (MAHA), thrombocytopenia, and microthrombi leading to tissue or organ injury [1]. This disorder can be further divided into primary or secondary forms. Primary forms occur spontaneously as in thrombotic thrombocytopenic purpura (TTP) or hemolytic uremic syndrome (HUS). Secondary forms usually have an associated underlying cause such as autoimmune disease, malignancy, pregnancy, malignant hypertension, or infection [1,2]. Not all TMAs will present with MAHA and thrombocytopenia and may be limited to the kidney. Timely treatment can prevent acute complications as well as the progression of the disease process [1-3].

This case was presented at the South Carolina American College of Physicians (ACP) Conference in October 2022.

## Case presentation

We present a 41-year-old male with a medical history of hypertension and hypothyroidism who presented with the chief complaint of worsening weakness and constipation. He reports that he was at his baseline state of health up until seven days prior to admission. Symptoms began with generalized weakness and malaise. Shortly after, he began to worsen, and symptoms were associated with nausea, vomiting, abdominal pain, and bilateral flank pain. On further questioning, he reported chronic headaches in which he would take a combination of acetaminophen 260 mg, aspirin 520 mg, and caffeine 32.5 mg packets frequently for many years. In the last week, he reported taking 6-7 packets a day for his pain with moderate symptomatic relief. The patient also reports that he was initially diagnosed with hypertension at the age of 16 and had been non-adherent to medications due to a lack of medical follow-up.

**Figure 1:**
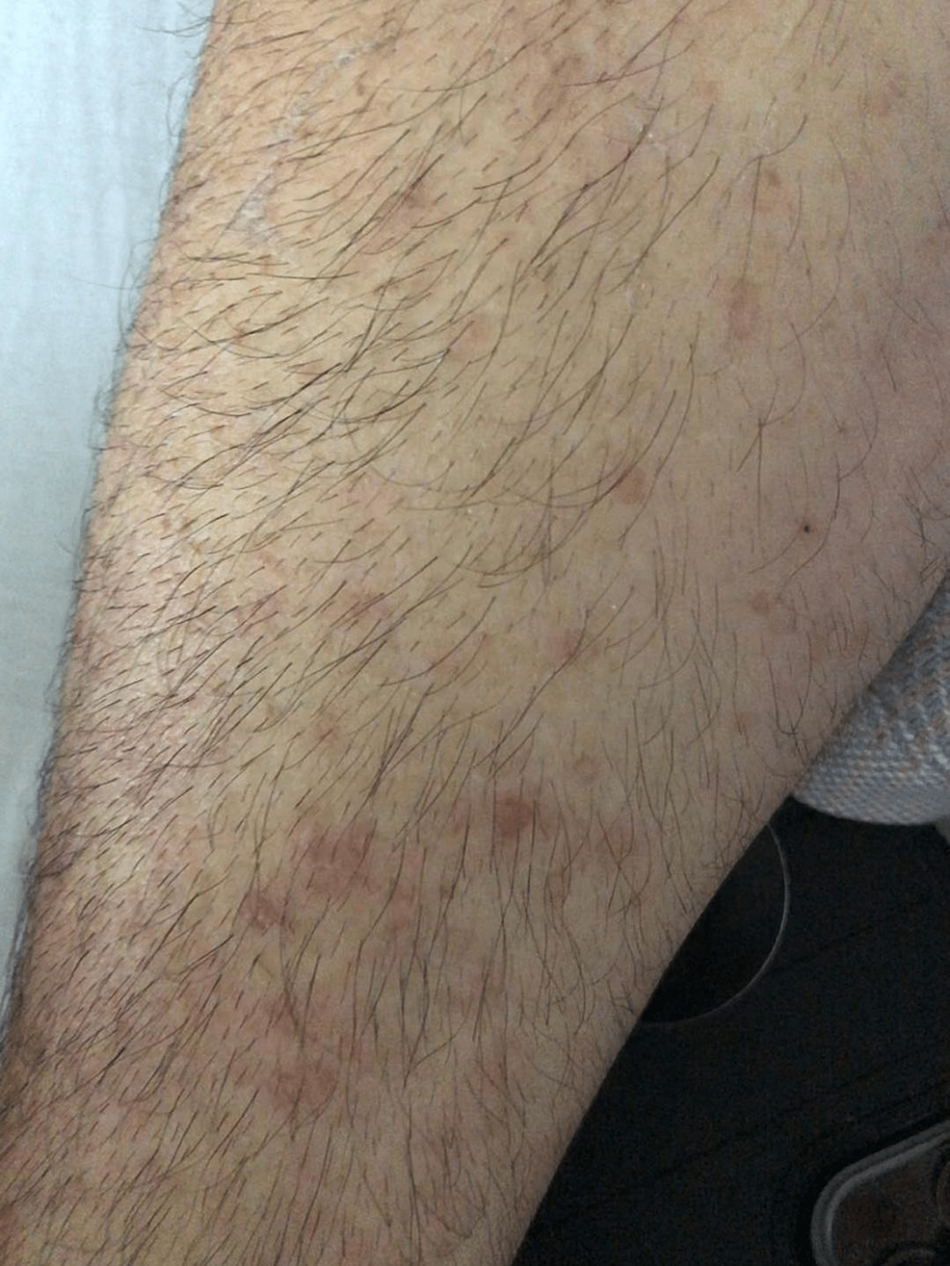
Diffuse rash This figure shows the patient’s right lower extremity. The rash was non-blanching and non-pruritic.

On initial presentation, his vitals were as follows: temperature of 98.4°F, pulse of 110 beats/minute, and blood pressure of 192/118. Physical examination was unremarkable, except for a diffuse upper and lower extremity rash that was non-blanching, flat, and non-pruritic (Figure 1). Initial laboratory results are summarized in Table 1. The laboratory results were significant for anemia and severe renal injury. CT of the abdomen and pelvis was done and unremarkable.

**Table 1:** Initial laboratory results This table shows the patient’s initial laboratory results upon presentation to our facility. The numbers in parentheses are the normal values/ranges. WBC: white blood cells; Hgb: hemoglobin; HCT: hematocrit; MCV: mean corpuscular volume; RDW: red cell distribution width; PLT: platelets; BUN: blood urea nitrogen; EST GFR: estimated glomerular filtration rate; CKD-EPI: Chronic Kidney Disease Epidemiology Collaboration; AST: aspartate transaminase; ALT: alanine transaminase; RBC: red blood cell; HPF: high-power field; LDH: lactate dehydrogenase; INR: international normalized ratio; PT: prothrombin time; PTT: partial thromboplastin time

Complete blood count	
WBC	16.2 K/mm^3^ (3.7-10.1 K/mm^3^)
Hgb	5 gm/dL (14-16.4 gm/dL)
HCT	14.7% (40%-47.2%)
MCV	89.6 fL (81.8-94.6 fL)
RDW	14.6% (11.6%-14%)
PLT count	142 K/mm^3^ (150-400 K/mm^3^)
Reticulocyte count	0.2158 mill/mm^3^ (0.02-0.11 mill/mm^3^)
Haptoglobin	<10 mg/dL (23-355 mg/dL)
Chemistry	
Sodium	124 mmol/L (136-145 mmol/L)
Potassium	4.5 mmol/L (3.5-5.1 mmol/L)
Chloride	81 mmol/L (98-107 mmol/L)
Carbon dioxide	21 mmol/L (21-32 mmol/L)
Anion gap	22 mEq/L (3-11 mEq/L)
BUN	142 mg/dL (7-18 mg/dL)
Creatinine	15.50 mg/dL (0.6-1.3 mg/dL)
EST GFR (CKD-EPI)	4 (≥60)
Glucose	120 mg/dL (74-106 mg/dL)
Calcium	8.5 mg/dL (8.5-10.1 mg/dL)
AST	40 units/L (15-37 units/L)
ALT	30 units/L (30-65 units/L)
Total protein	6.4 gm/dL (6.4-8.2 gm/dL)
Albumin	2.9 gm/dL (3.5-5 gm/dL)
LDH	866 units/L (100-190 units/L)
Total bilirubin	0.5 mg/dL (0.2-1 mg/dL)
Urinalysis	
pH	6.0 (5.0-8.5)
Urine protein	500 mg/dL (negative)
RBC	>100 RBC/HPF (0-1 RBC/HPF)
WBC	0-1 WBC/HPF (0-1 WBC/HPF)
Nitrite	Negative
Leukocyte esterase	Negative
Coagulation panel	
INR	1.28 (0.9-1.1)
PT	14.5 seconds (9.8-13.9 seconds)
PTT	33.6 seconds (24.9-37.9 seconds)

Due to the patient’s symptomatic anemia, he was transfused with 1 unit of packed red blood cells. Blood pressure medications were titrated to improve hypertension. Symptoms improved; however, the patient became anuric, and a shared decision with the patient was made to start hemodialysis.

On further workup, the peripheral blood smear showed absolute granulocytosis with anemia, and no schistocytes were noted. Cytomegalovirus (CMV), HIV, hepatitis A, B, and C, anti-DNase B, and mycoplasma antibodies were negative. Epstein-Barr virus (EBV) IgM antibody was negative, and EBV IgG antibody was positive. Parathyroid hormone (PTH) was 543 pg/mL (normal values: 18.5-88 pg/mL), alpha-2 globulin was 0.7 g/dL (normal values: 0.4-1 g/dL), beta globulin was 0.7 g/dL (normal values: 0.4-1 g/dL), and gamma globulin was 0.4 g/dL (normal values: 0.4-1.8 g/dL). No M-spike was observed in serum protein electrophoresis. His Coombs test was negative, as well as his antiphospholipid antibody. IgG, IgA, IgM, antinuclear antibody (ANA), rheumatoid factor, cytoplasmic antineutrophil cytoplasmic antibodies (c-ANCA), perinuclear antineutrophil cytoplasmic antibody (p-ANCA), Sjögren’s syndrome-related antigen A (SS-A), SS-B, kappa/lambda, and complement C3 and C4 were unremarkable. A PLASMIC score was calculated with a yield of 4 (evidence of hemolysis, no history of active cancer, no history of solid organ or stem cell transplant, and INR < 1.5), which demonstrated low suspicion for TTP requiring emergent plasmapheresis. An ADAMTS13 was obtained showing activity of 70.5%. Ultrasound of the kidneys was performed, and the results demonstrated medical renal disease with no evidence of obstruction.

**Figure 2:**
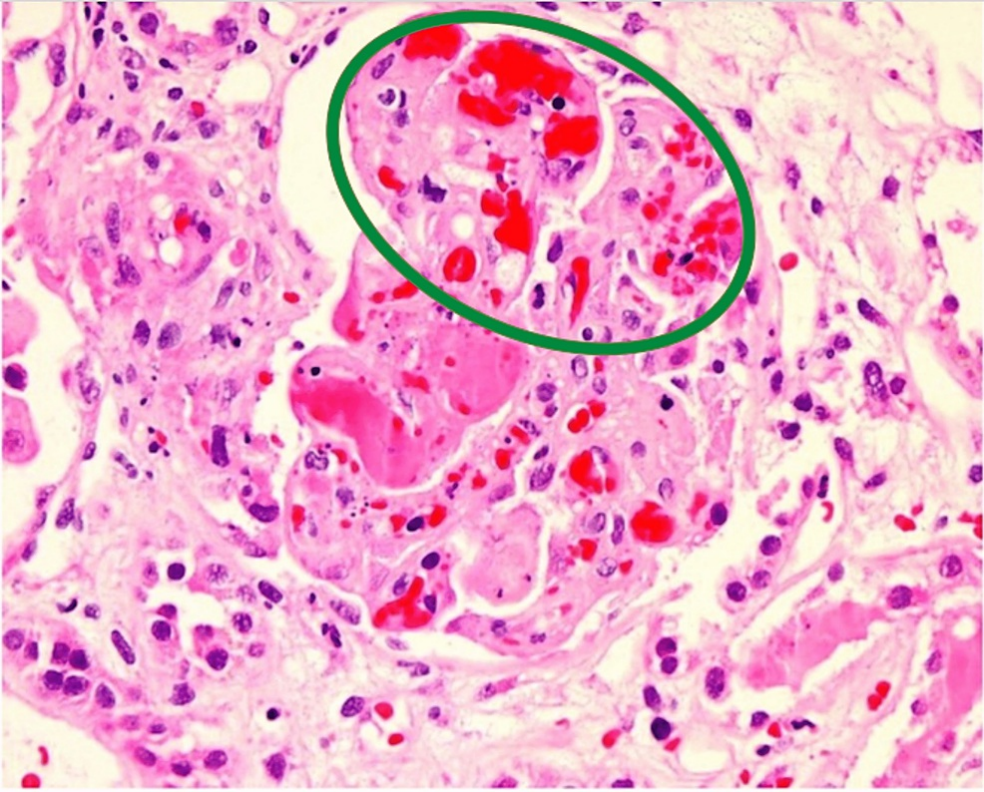
Right renal biopsy showing thrombotic microangiopathy In this figure, the green circle encompasses the thrombotic microangiopathy (TMA) that is affecting an estimated 20% of the glomeruli.

In addition to the laboratory work, a renal biopsy was done, which resulted in thrombotic microangiopathy affecting 20% of the glomeruli (Figure 2). He was diagnosed with hypertension-induced thrombotic microangiopathy. His symptoms improved, and a permanent catheter was placed for further dialysis.

## Discussion

Thrombotic microangiopathies (TMAs) are a group of life-threatening hematological conditions that usually present with microangiopathic hemolytic anemia (MAHA), thrombocytopenia, and microthrombi leading to tissue or organ injury. This clinical pathology can be further divided into primary or secondary forms. Primary forms occur spontaneously as in thrombotic thrombocytopenic purpura (TTP) or hemolytic uremic syndrome (HUS). Secondary forms usually have an associated underlying cause such as autoimmune disease, malignancy, pregnancy, malignant hypertension, or infection [1,2]. Not all TMAs will present with MAHA and thrombocytopenia and may be limited to the kidney [1-3]. Suspicion of thrombotic microangiopathy should be considered in patients with anemia, thrombocytopenia, and renal injury. However, as TMAs can occur in multiple clinical settings with various pathological processes, it is essential that the underlying cause be identified quickly as treatment varies based on the underlying pathology.

Hypertension is a major cause of morbidity and mortality worldwide. An estimated 3% of all emergency visits and 25% of all medical emergencies can be attributed to hypertensive urgency [3]. Although this is common, it is rare for these episodes to cause isolated thrombotic microangiopathy in the kidney [3-6].

The initial evaluation is focused on confirming MAHA and thrombocytopenia and ruling out systemic disorders that may manifest in similar ways. Some of these disorders are TTP, complement-mediated TMA, Shiga toxin-mediated HUS, disseminated intravascular coagulation (DIC), infections, and severe hypertension. Clinical features may assist in distinguishing between TMAs. Ingestion of undercooked meat, fever, gastrointestinal symptoms, and the severity of thrombocytopenia can help guide the diagnostic process [1-3].

Helpful laboratory tests when suspecting TMA and assisting in monitoring disease progression include daily complete blood count, lactate dehydrogenase, and serum creatinine. ADAMTS13 activity should be measured; however, if there is a high suspicion for TTP, it is not recommended to wait for results or to start treatment. Since TTP is a disorder with the greatest mortality, therapeutic plasma exchange should be initiated when there is high suspicion [1-3].

As in our case, significant renal impairment, severe hypertension, relatively normal platelet count, and lack of ADAMTS13 deficiency (activity > 10%) do not fit the classic pentad of TTP or HUS. A kidney biopsy may be needed to pinpoint the disease process since it may be essential to distinguish TMA from other causes of renal injury. However, it may not be helpful in distinguishing TMA syndromes [1,2].

The inability to reliably differentiate TTP from other etiologies presents a dilemma for clinicians as treatment depends on the underlying pathology. In hypertension-induced TMA, plasma exchange is not utilized, and strict blood pressure control is the treatment of choice. If diagnosed early enough, renal function may be preserved and possibly improved. Unfortunately, in some cases, severe hypertension may lead to irreversible renal injury requiring dialysis [2,3].

## Conclusions

In conclusion, we present an interesting case of a young patient with uncontrolled hypertension leading to a secondary form of thrombotic microangiopathy isolated to the kidney. This TMA syndrome can mimic TTP or other physiologies of microangiopathy, which makes the cause of the pathology difficult to discern. The treatment of hypertension-induced thrombotic microangiopathy is blood pressure control versus its counterparts that may require plasma exchange. We shed light on the diagnosis and management of a patient with TMA in the absence of classical findings.
